# Weight Status, Adherence to the Mediterranean Diet, Physical Activity Level, and Sleep Behavior of Italian Junior High School Adolescents

**DOI:** 10.3390/nu12020478

**Published:** 2020-02-13

**Authors:** Alice Rosi, Francesca Giopp, Giulia Milioli, Gabriele Melegari, Matteo Goldoni, Liborio Parrino, Francesca Scazzina

**Affiliations:** 1 Department of Food and Drugs, University of Parma, 43125 Parma, Italy; alice.rosi@unipr.it (A.R.); francesca.giopp@studenti.unipr.it (F.G.); gabriele.melegari@studenti.unipr.it (G.M.); 2 Department of Medicine and Surgery, University of Parma, 43126 Parma, Italy; giulia.milioli@unipr.it (G.M.); matteo.goldoni@unipr.it (M.G.); liborio.parrino@unipr.it (L.P.)

**Keywords:** adolescence, body mass index, KIDMED score, physical activity level, sleep habits, sleep duration, students, young, school achievement, school performance

## Abstract

Inadequate diet, physical activity, and sleep-related behaviors are potential risk factors for overweight and obese, therefore we investigated the relations between body mass index (BMI) and behavioral factors in a sample of Italian adolescents. Four hundred nine Italian secondary school students (46% females, 12.5 ± 0.6 y.o.) were enrolled in this cross-sectional study. Anthropometric measures, adherence to the Mediterranean Diet (KIDMED), physical activity level (PAQ-C), sleep duration, daytime sleepiness (PDSS), sleep quality, and school achievement data were collected through an online questionnaire. The percentage of overweight adolescents was slightly lower (14%) compared to the regional and the national figures. Approximately 88% of the sample reported a medium/high adherence to the Mediterranean Diet and 77% a moderate/vigorous physical activity level. The average sleep duration was in line with the international sleep recommendation for adolescents and 82% had a medium/high sleep quality. No differences were found between genders except for BMI (lower in females). Unexpectedly, no differences were found among the BMI groups (normal weight vs. overweight vs. obese) for lifestyle variables; in contrast, Mediterranean Diet adherence was associated with sleep habits. Further investigation is required to better explore the associations among behavioral variables involved in adolescents’ healthy development.

## 1. Introduction

In the past decades, critical modifications of adolescents’ lifestyle have been observed in Europe, leading to the alarming rise in obesity and to the relevant incidence of chronic non-communicable diseases from childhood to adulthood [1]. Among Mediterranean countries, Greece has shown the highest percentage of overweight and obese adolescents aged 15 years (21.5%); higher values have been reported only out of Europe (Canada: 24.5% and United States: 31%), while Italy results in line with the European average (16%) [2].

Several factors have been investigated in the etiology of obesity: first of all, changing in dietary patterns [3]. During childhood and adolescence, a balanced diet promotes optimal health, growth, and cognitive development and may contribute, later in life, to the prevention of chronic diseases [4,5]. Unfortunately, a fast and global spread of highly processed products and ready-to-eat products has been observed worldwide, mainly because of the globalization of the food sector [3,6,7]. Consequently, a gradual shift from the dietary behavior of the Mediterranean Diet (MD)—rich in fruit and vegetables, whole grain cereals, fish, and olive oil—toward a Western Diet, characterized by high intake of red and processed meat, pre-packaged foods, high-fat dairy products and refined grains, has been observed also in Mediterranean countries and could be enumerated among the factors leading to an excessive calorie intake [8,9]. Furthermore, the reduction of physical activity and time spent playing outside has been recognized as an additional risk factor for obesity and several chronic diseases [10]. In addition, screen media exposure has also been linked to the risk of being obese through increased eating, since a higher exposure to high-calorie, low-nutrient food, and beverage marketing could influence adolescents’ food preferences, purchase, and consumption [11]. Along with diet and physical activity, insufficient sleep may negatively affect the body weight [12,13]. In the past decades, chronic sleep deprivation has been reported among adolescents, due to modern lifestyle, the use of artificial light and electronic devices during the night, as well as lack of sleep hygiene in the family context [14]. Furthermore, sleep deprivation and late bedtimes increase the opportunities for eating and may lead to reduction of physical activity because of fatigue and tiredness [15]. The combined effect of all these variables has been associated not only with adolescents’ weight status but also with school achievement and cognitive performance [16]. Healthy lifestyle behaviors do not act in isolation and the effects of multiple healthy lifestyle behaviors may be greater than the sum of their individual impact. In view of these considerations, investigating adolescent’s lifestyle appears to be fundamental not only to monitor the incidence of overweight and obesity in teenagers but also to define the relative contribution of lifestyle habits (e.g., diet, physical activity, and sleep) on the etiology of overweight and obesity in youth. In this framework, the school setting can be an appropriate environment for assessing lifestyle, modifying misconducts, and educating students, while maintaining a stable contact with them.

In keeping this evidence, we hypothesized that adolescents’ weight status could be associated with their lifestyle and behaviors, such as diet, physical activity, and sleep habits. Therefore, we carried out an observational study in a convenient sample of junior high school students living in Parma (Italy), with the aim to investigate associations between weight status and adherence to the MD, physical activity level, sleep quantity and quality, and school achievement. In addition, associations between diet and physical activity, sleep habits, and school performance were investigated to better understand the relation between diet and lifestyle factors.

## 2. Materials and Methods

### 2.1. Participants and Study Design

This cross-sectional study was carried out during 2016–2017 and 2017–2018 school years in the city of Parma (North Italy). Male and female public junior high school students, aged between 11 and 14 years old, were asked to participate in this study. Exclusion criteria were mental or intellectual disability, serious physical disability, and eating disorders. Students and parents/legal guardians were informed about the aim and the methodology of the project, they were guaranteed of complete anonymity and they were both asked to give their written informed consent.

The study was performed according to the Declaration of Helsinki and was approved by the Institutional Review Board (IRB) of the University of Parma and by the School Board and the School Principal of the two schools.

For each student data were collected on the same day during the school hours by administering a self-report online questionnaire. Compilation of the questionnaires was supported by trained researchers.

#### 2.1.1. Personal Data

Age, sex, body weight, and height were self-reported by each student. Body max index (BMI) was calculated and used to define the weight status through the IOTF (International Obesity Task Force) gender- and age-related cut-offs for BMI [17].

#### 2.1.2. Adherence to the Mediterranean Diet (MD)

Adherence to the MD was assessed through the KIDMED test (Mediterranean Diet Quality Index for children and teenagers) [18], based on 16 yes/no questions on dietary habits representative or not representative of the MD. A score of −1 was assigned to the answers whose questions denoted a negative connotation with respect to the MD, while +1 point was assigned to the answers whose questions represented a Mediterranean habit. The total KIDMED score ranged between 0 and 12 points, and it was classified into three levels of adherence to the MD: high adherence (score ≥ 8 points); medium adherence (score 4–7 points) and low adherence (score ≤ 3 points).

#### 2.1.3. Physical Activity

The physical activity level of students was investigated using the PAQ-C questionnaire (Physical Activity Questionnaire for older Children), a validated questionnaire self-administered to assess the level of physical activity of 8–14 years old respondents, referred to the 7 days before compilation [19]. The PAQ-C was composed of nine questions on several physical activities, scored on a 5-point scale, plus a last question aimed to understand whether the described week was representative of a normal week or if something had occurred changing the regular physical activity level. On the basis of the given answers, students were classified as sedentary, low active, active, very active, or not having a regular week activity.

#### 2.1.4. Sleep Behavior

Sleep duration was calculated as the difference between self-reported bedtime in the evening and wake up time in the morning, and it was differentiated between school days and weekend days. The average week sleep duration was calculated in hours as: (weekday sleep duration × 5 + weekend sleep duration × 2)/7. According to the National Sleep Foundation recommendations for school-age children and teenagers [20], sleep duration was classified as low if less than 8 h per night, recommended if between 8 and 11 h per night or high if more than 11 h per night.

Daytime sleepiness was assessed through the PDSS questionnaire (Pediatric Daytime Sleepiness Scale), a validated tool for assessing self-reported sleepiness during the day of students aged between 11 and 15 years [21]. Through eight multiple-choice questions it allows to define sleepiness based on the perception of drowsiness and tiredness. A score from 0 to 4 points is assigned to each question, and the final PDSS total score ranges between 0 to 32 points, with higher values corresponding to higher daytime drowsiness.

Sleep quality was indirectly defined using the total PDSS score, since a high daytime sleepiness could be a consequence of poor sleep quality. Students with a total PDSS score ≤ 10 points were considered as having a high sleep quality, those with a score between 11 and 20 as having a medium sleep quality, and the ones with a score > 20 points as having a low sleep quality.

#### 2.1.5. School Performance

School achievement was assessed considering the average grade of the current school year as a range between 0 (very poor) and 10 (outstanding) and it was transformed in a categorical variable as excellent (average grade ≥ 9), good (average grade between 7 and 8), and poor (average grade ≤ 6).

### 2.2. Statistical Analysis

Junior high school students in Parma were around 4500 at the running period of the study. Keeping 95% level of confidence and 5% marginal error, the study population should was composed of 354 adolescents. To reach a convenient sample, around 500 students were invited to participate in the study considering that some students could not obtain parents/legal guardians informed consent or could not give their informed consent and that some participants were excluded from the analyses because of the absence during the assessment day, missing values, or wrong-reported data.

The Statistical Package for Social Science SPSS version 25.0 (SPSS Inc., Chicago, IL, USA) was used to perform all the statistical analyses, establishing the significance at *p* < 0.05. Data were expressed as means ± standard deviations (SD) for continuous variables or as numbers/percentages for categorical variables. The normality of distribution was evaluated through the Kolmogorov-Smirnov test. The Student t-test was used to compare continuous variables between genders (males vs. females). Differences among BMI groups (normal weight vs. overweight vs. obese) or adherence to the MD groups (low vs. medium vs. high adherence) were tested performing a one-way ANOVA with Bonferroni post hoc test, after assessing the equality of variance by using the Levene’s test. Moreover, a one-way ANCOVA was carried out searching for differences among BMI groups after adjusting for sex. A Pearson chi-square test was used to compare categorical variables between genders and among BMI groups and adherence to the MD groups. Finally, a linear stepwise regression was performed to explore the independent variables (sex, age, KIDMED score, PAL, average sleep time, and PDSS score) involved in predicting BMI (dependent variable), setting the stepping method criteria to be taken at *p* < 0.10 and to be removed at *p* > 0.15.

## 3. Results

### 3.1. Adolescents’Anthropometrics and Lifestyle Factors

Out of 500 potentially eligible students, 448 received parents/legal guardians’ informed consent and were in line with the inclusion and exclusion criteria. As 39 students were absent from school during the assessment day, a total of 409 adolescents, 222 males (54%), and 187 females (46%) completed the study (response rate 91%). Data collected from the whole sample and divided by gender are presented in Table 1.

No differences were found between males and females for both weight and height, while the BMI was significantly lower in females than in males (*p* = 0.004). Moreover, BMI distribution into underweight, normal weight, and overweight categories was different between genders, showing an association between these two variables (X^2^ = 12.26, df = 2, *p* = 0.002). The average KIDMED score corresponded to a medium adherence to the MD and was similar between genders. Regarding the PAL, 80 participants (20%) declared to have not performed a regular physical activity during the previous week (to which the questionnaire was referred) because of physical problems or additional school activities. To avoid results not representative of the real PAL of the participants, physical activity data were analyzed only in students reporting regular activities during the previous week, and the PAL was found to be not associated with gender. Considering the whole week, the total sleep duration reached almost 9 h per night, corresponding to the recommended sleep duration for junior high school students, while the total mean PDSS score corresponded to a medium sleep quality. Sleep duration, daytime sleepiness, and sleep quality were found to be similar between genders. No association was found also between school achievement and gender.

To explore the possible behavioral variables responsible for the students’ BMI, participants were grouped by their weight status (Table 2).

No differences among BMI groups were found for all the lifestyle variables, not even when adjusted for sex (*p* > 0.05 for all variables). Moreover, weight status was not associated with adherence to the MD, PAL, sleep duration adequacy, sleep quality, and school achievement.

The stepwise regression analysis revealed that sex (B = 0.813, SE = 0.298, beta = 0.149, t = 2.731, *p* = 0.007), KIDMED score (B = −0.133, SE = 0.065, beta = −0.112, t = −2.045, *p* = 0.042), and average sleep time (B = −0.323, SE = 0.187, beta = −0.094, t = −1.725, *p* = 0.086) were predictors of BMI but this model explained merely 4% of the variance (*F*(3, 321) = 4.892, *p* = 0.002).

Lastly, students were grouped by their adherence to the MD, to better explore the association between diet and other lifestyle habits (Table 3).

No significant associations were found between adherence to the MD and PAL. However, adherence to the MD was associated with sleep duration (X^2^ = 11.15, df = 4, *p* = 0.025), with sleep duration found to be adequate in the medium and high adherence to the MD groups. In addition, significant differences among adherence to the MD categories and daytime sleepiness were reported (*p* = 0.005) with higher drowsiness (higher PDSS scores) found in the students who had a lower adherence to the MD. An association was also observed between adherence to the MD and sleep quality categories (X^2^ = 13.21, df = 4, *p* = 0.010). School achievement was found to be different among adherence to the MD groups (*p* = 0.018), with the high adherence group having an academic performance greater than the low adherence group, but the two categorical variables were not associated.

### 3.2. Dietary Habits

**Figure 1:**
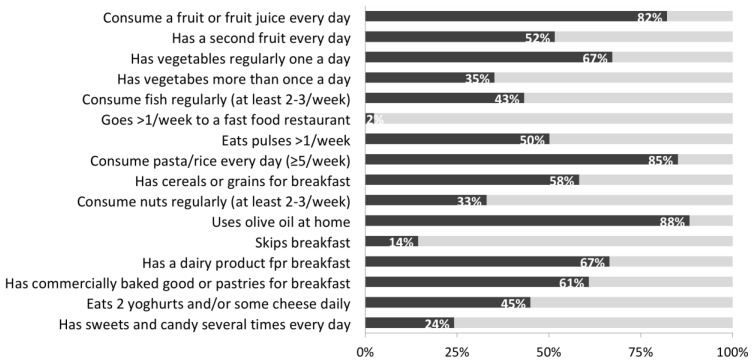
Responses to the KIDMED questionnaire (Mediterranean Diet Quality Index in Children and Adolescents) for the total sample.

The responses to each single question of the KIDMED questionnaire for the total sample are presented in Figure 1. Fruit and fruit juices were consumed daily by 82% of participants, 52% had a second portion every day, and 33% ate nuts regularly. In addition, 67% of students ate vegetables each day and 35% consumed vegetables more than once a day. In relation to protein-based food, 43% of participants ate fish regularly and 50% had pulses more than once a week. Pasta or rice was consumed almost every day by 85% of children. Breakfast was regularly reported by 86% of students, of these 58% consumed cereals or grains, 67% milk or dairy products, and 61% commercially baked goods or pastries for breakfast. In addition, 46% ate two yoghurts and/or cheese daily, and 88% used olive oil as main fat condiment when eating at home. Only 2% of the sample went more than once per week to a fast food restaurant, and 24% had sweets and/or candy several times every day.

## 4. Discussion

This study provides an overview of the relationship between weight status and behavioral factors in a substantial sample of junior high school Italian adolescents. Contrary to what was expected, adolescents’ weight status was not associated with PAL and daytime sleepiness, while sex, adherence to the MD, and average sleep duration were predictors of adolescents’ BMI.

Regarding body composition, 14% of participants were overweight or obese, a slightly lower percentage compared to the one recorded in the Emilia Romagna region (15%) (where the city of Parma is located), and to national data (16%) for 11–15-year-old adolescents enrolled in the HBSC study [22,23]. A higher discrepancy was observed for underweight (17%) and normal weight (69%) adolescents in this study, compared to the national data characterized by 3% of underweight adolescents and around 81% of the normal weight adolescents [22,23]. In addition, a higher prevalence of overweight and obese male adolescents compared to females was observed, as widely confirmed in literature for Italian and other European populations [22,23,24,25,26,27,28,29]. BMI was found to be associated with sex but not with physical activity, as previously reported in primary school children living in the same area [25].

Besides sex, adolescents’ BMI was predicted by adherence to MD. Most subjects (60%) had a medium adherence to the MD, in line or slightly higher compared to recent results on Italian and Spanish adolescents [27,30,31]. Fruit and vegetable frequencies of consumption were similar to the regional and national data [22,23]. Moreover, less than half of students ate fish regularly, only 50% had legumes more than once a week, and 24% consumed sweets several time every day, in line with the results presented in a recent study on adolescent adherence to the MD in Spain [30]. These habits should be improved through specific educational programs aiming to improve fish, legume, and nut intakes, which were under the recommended value according to the national data [32]. It is also important to underline that only 14% of participants were breakfast skippers, a lower percentage compared to the figures observed in Italian and foreign populations, where percentages were around 20% [22,23,33,34].

In the present study, no evidence was found on associations between adherence to the MD and gender. The associations between adherence to the MD and gender, and adherence to the MD and BMI are not well established in adolescents [31]. Associations between these variables were not found in some Italian and European investigations [27,35,36], but in a multicenter study on Italian adolescents, overweight status and obesity were higher in participants having a lower adherence to the MD compared to the normal weight adolescents [37]. Similarly, adherence to the MD and PAL were not associated, in disagreement with the more recent literature evidence reporting a robust positive association between these two variables [31]. On the contrary, interesting associations were observed between adherence to the MD and sleep behaviors. Good sleep habits have been related to healthier food patterns [38] and higher adherence to the MD [31], while alterations in sleep quality (sleep patterns and sleep efficiency) have often been associated with unhealthy habits and lifestyle modifications, such as lower physical activity and consumption of high calorie foods and beverages, far from the characteristics of the Mediterranean pattern [39,40,41]. Lastly, students with a high adherence to the MD had better school achievements, in agreement with other European studies [25,31,42].

The majority of the students claimed to accomplish adequate PAL, which reflects literature data from foreign and Italian surveys [22,43]. A positive association was observed between physical activity and higher school grades as already described by Coe and colleagues [44], but disagreed with Rasberry and colleagues [45] who found no significant association between these two variables No association was also found between physical activity levels and other variables in this study, although in literature a conspicuous body of evidence revealed a gender difference in physical activities, with females usually performing less sports and general physical activities compared to males [23,46].

Considering sleep behavior, the majority of students declared to habitually sleep the recommended 8–10 h per night, according to the American Academy of Sleep Medicine [47] and to the National Sleep Foundation recommendations [20]. Besides the positive association found between sleep duration and adherence to the MD, sleep length was also associated to better school performances, in line with several cross-sectional investigations described in a systematic review on adolescents sleep habits [48] and with findings from the HELENA study in a European young population [49]. Moreover, average sleep duration was a predictor of adolescent BMI. In literature, a direct association between poor sleep duration and overweight-obesity in both children and adults has also been documented [50]. In particular, short sleep duration increased the risk of childhood obesity [13] and was found to be associated with subsequent overweight/obesity in longitudinal studies with young subjects [12,51]. Among European adolescents, shorter sleepers showed higher values of BMI, probably as a consequence of a not balanced energy intake mainly because of sedentary habits and excessive food consumption [40].

The main limitation of this study is the use of self-reported data, such as body weight and height, dietary habits, physical activity level, sleep behaviors, and academic performance. Nevertheless, the technological platform applied appeared to be effective in collecting behavioral data through online questionnaires, improving the compliance and limiting the missing data. Adolescents’ lifestyle behaviors could also be associated with parental habits, socio-economic level, and other personal characteristics like the Tanner stage and the menarche stage, which were not investigated in the present study. Furthermore, other behavioral factors including screen time, media exposure and use of electronic devices should be further explored, in particular before sleeping. Moreover, the study population could not be considered representative of Italian adolescents and further investigations are recommended, involving larger and multi-center populations. Lastly, a point worth of attention is the fact that cross-sectional designs prevent to derive causal relationships between variables. However, this study identifies critical behaviors in a sample of Italian junior high school students, providing suggestions on future educational programs, which should be based on prevention of overweight and obesity and lifestyle improvement among the new generations.

In summary, this study gives a detailed insight of the lifestyle of junior high school adolescents living in Parma (North Italy). Weight status was not associated with physical activity level and sleep quality. However, it should be in mind that approximately two-third of our study population showed a normal weight status and reported a medium-high adherence to the MD, a moderate-vigorous physical activity level, and an adequate sleep duration and sleep quality. This overall positive situation may mask the potential associations of these behavioral variables with adolescents’ BMI. From our study, a better adherence to the MD was positively associated with most of the behavioral variables considered, stressing the primary role of this dietary pattern in adolescent wellbeing. Nevertheless, much still needs to be done to improve adherence to the MD in adolescent populations, for example, promoting fish and legumes intake and discouraging sweets and candies consumption.

Further studies are required to better explore the whole lifestyle habits in adolescent populations and identify the behavioral factors involved in the onset of overweight and obesity. The identification of the priorities on which it would be necessary to act would allow involving all those realities around adolescents (e.g., family, school, sport associations) to develop synergic actions useful in the continuous improvement of healthy lifestyle for young people through a multidisciplinary approach.

## 5. Conclusions

This study showed that adolescents’ BMI can be predicted by adherence to the MD and sleep quantity, thus improving the dietary habits, and assuring adequate sleep duration among adolescents may play a key role in the prevention of youth obesity.

## Figures and Tables

**Table 1:** Anthropometric measures and lifestyle data for the total samples and by gender.

Variables	Total Sample (*n* = 409)	Male (*n* = 222)	Female (*n* = 187)	*p* Value
Age (years)	12.5 ± 0.6	12.5 ± 0.6	12.5 ± 0.7	0.759
Weight (kg)	44.9 ± 9.1	45.7 ± 9.2	43.9 ± 8.9	0.055
Height (m)	1.6 ± 0.1	1.6 ± 0.1	1.6 ± 0.1	0.888
BMI (kg/m^2^)	18.4 ± 2.7	18.7 ± 2.6	18.1 ± 2.8	0.014
BMI category				0.004
Underweight	67 (16%)	25 (11%)	42 (22%)	
Normal weight	284 (70%)	159 (72%)	125 (67%)	
Overweight	58 (14%)	38 (17%)	20 (11%)	
KIDMED score (points)	6.0 ± 2.3	6.0 ± 2.3	6.1 ± 2.3	0.857
Adherence to the MD				0.979
Low	51 (12%)	27 (12%)	24 (13%)	
Medium	244 (60%)	133 (60%)	111 (59%)	
High	114 (28%)	62 (28%)	52 (28%)	
Physical Activity Level				0.938
Sedentary	2 (0%)	1 (1%)	1 (1%)	
Light	76 (23%)	38 (22%)	38 (25%)	
Moderate	206 (63%)	110 (63%)	96 (62%)	
Vigorous	45 (14%)	25 (14%)	20 (13%)	
Week sleep time (hh:mm)	8:33 ± 00:49	8:29 ± 00:50	8:38 ± 00:46	0.640
Weekend sleep time (hh:mm)	9:55 ± 01:31	9:51 ± 01:27	10:00 ± 01:35	0.892
Average sleep time (hh:mm)	8:56 ± 00:50	8:52 ± 00:51	9:01 ± 00:49	0.801
Average sleep time adequacy				0.464
Low	55 (13%)	32 (14%)	23 (12%)	
Adequate	352 (86%)	190 (86%)	162 (87%)	
High	1 (0%)	0 (0%)	1 (1%)	
PDSS score (points)	15.8 ± 5.7	15.3 ± 5.9	16.4 ± 5.4	0.072
Sleep quality				0.526
Low	88 (22%)	43 (20%)	45 (24%)	
Medium	244 (60%)	136 (62%)	108 (58%)	
High	74 (18%)	41 (19%)	33 (18%)	
School average (grade)	7.8 ± 1.0	7.8 ± 1.0	7.8 ± 1.1	0.744
School performance				0.373
Excellent	103 (25%)	52 (23%)	51 (27%)	
Good	268 (65%)	152 (68%)	116 (62%)	
Poor	38 (9%)	18 (8%)	20 (11%)	

Data are presented as mean ± SD for continuous variables and frequency (% of total sample) for categorical variables; BMI: body mass index; KIDMED: Mediterranean Diet Quality Index in Children and Adolescents; MD: Mediterranean Diet; PDSS: Pediatric Daytime Sleepiness Scale.

**Table 2:** Relations between BMI groups and adherence to the MD, PAL, sleep habits, and school performance.

Variables	Total Sample (*n* = 409)	Underweight (*n* = 67)	Normal Weight (*n* = 284)	Overweight (*n* = 58)	*p* Value
KIDMED score (points)	6.0 ± 2.3	6.2 ± 2.4	6.0 ± 2.1	5.8 ± 2.7	0.569
Adherence to the MD					0.459
Low	51 (12%)	7 (10%)	33 (12%)	11 (19%)	
Medium	244 (60%)	39 (58%)	175 (62%)	30 (52%)	
High	114 (28%)	21 (31%)	76 (27%)	17 (29%)	
Physical Activity Level					0.313
Sedentary	2 (0%)	1 (1%)	1 (0%)	0 (0%)	
Light	76 (23%)	11 (22%)	50 (22%)	15 (31%)	
Moderate	206 (63%)	32 (63%)	150 (66%)	24 (49%)	
Vigorous	45 (14%)	7 (14%)	28 (12%)	10 (20%)	
Week sleep time (hh:mm)	08:33 ± 00:49	08:36 ± 00:49	08:34 ± 00:47	08:22 ± 00:55	0.194
Weekend sleep time (hh:mm)	09:55 ± 01:31	09:58 ± 01:48	09:56 ± 01:24	09:44 ± 01:41	0.648
Average sleep time (hh:mm)	08:56 ± 00:50	08:59 ± 00:56	08:57 ± 00:48	08:45 ± 00:55	0.224
Average sleep time adequacy					0.696
Low	55 (14%)	9 (13%)	35 (12%)	11 (19%)	
Adequate	352 (86%)	58 (87%)	247 (87%)	47 (81%)	
High	1 (0%)	0 (0%)	1 (0%)	0 (0%)	
PDSS score (points)	15.8 ± 5.7	16.0 ± 5.8	15.8 ± 5.5	15.7 ± 6.3	0.959
Sleep quality					0.880
Low	88 (22%)	16 (24%)	58 (21%)	14 (25%)	
Medium	244 (60%)	41 (61%)	171 (61%)	32 (56%)	
High	74 (18%)	10 (15%)	53 (19%)	11 (19%)	
School average (grade)	7.8 ± 1.0	7.9 ± 0.9	7.8 ± 1.0	7.6 ± 1.0	0.302
School performance					0.498
Excellent	103 (25%)	15 (22%)	75 (26%)	13 (22%)	
Good	268 (66%)	49 (73%)	180 (63%)	39 (67%)	
Poor	38 (9%)	3 (5%)	29 (10%)	6 (10%)	

Data are presented as mean ± SD for continuous variables and frequency (% of total sample) for categorical variables; MD: Mediterranean Diet; PAL: physical activity level; KIDMED: Mediterranean Diet Quality Index in Children and Adolescents; PDSS: Pediatric Daytime Sleepiness Scale.

**Table 3:** Relations between adherence to the MD and PAL, sleep habits, and school performance.

	Adherence to the Mediterranean Diet				
Variables	Total Sample (*n* = 409)	Low (*n* = 51)	Medium (*n* = 244)	High (*n* = 114)	*p* Value
Physical activity level					0.135
Sedentary	2 (0%)	1 (3%)	1 (1%)	0 (0%)	
Light	76 (23%)	8 (21%)	51 (26%)	17 (18%)	
Moderate	206 (63%)	27 (71%)	117 (60%)	62 (64%)	
Vigorous	45 (14%)	2 (5%)	25 (13%)	18 (19%)	
Week sleep time (hh:mm)	08:33 ± 00:49	08:43 ± 00:47	08:30 ± 00:50	08:35 ± 00:47	0.138
Weekend sleep time (hh:mm)	09:55 ± 01:31	09:56 ± 01:46	09:53 ± 01:28	09:57 ± 01:30	0.471
Average sleep time (hh:mm)	08:56 ± 00:50	09:04 ± 00:56	08:54 ± 00:51	08:59 ± 00:47	0.134
Average sleep time adequacy					0.025
Low	55 (13%)	10 (20%)	35 (14%)	10 (9%)	
Adequate	352 (86%)	40 (78%)	208 (86%)	104 (91%)	
High	1 (0%)	1 (2%)	0 (0%)	0 (0%)	
PDSS score (points)	15.8 ± 5.7	17.4 ± 5.4 ^a^	16.1 ± 5.7 ^ab^	14.5 ± 5.4 ^b^	0.005
Sleep quality					0.010
Low	88 (22%)	17 (33%)	58 (24%)	13 (12%)	
Medium	244 (60%)	29 (57%)	142 (58%)	73 (65%)	
High	74 (18%)	5 (10%)	43 (18%)	26 (23%)	
School average (grade)	7.8 ± 1.0	7.5 ± 1.0 ^b^	7.8 ± 1.0 ^ab^	8.0 ± 1.0 ^a^	0.018
School performance					0.083
Excellent	103 (25%)	9 (18%)	58 (24%)	36 (32%)	
Good	268 (66%)	33 (65%)	165 (68%)	70 (61%)	
Poor	38 (9%)	9 (18%)	21 (9%)	8 (7%)	

Data are presented as mean ± SD for continuous variables and frequency (% of total sample) for categorical variables; Different letters in the same row indicate significant differences among adherence to the MD groups (one-way ANOVA with Bonferroni post hoc test); MD: Mediterranean Diet; PAL: Physical Activity Level; PDSS: Pediatric Daytime Sleepiness Scale.
